# Carbohydrate intake quality and gestational diabetes mellitus, and the modifying effect of air pollution

**DOI:** 10.3389/fnut.2022.992472

**Published:** 2023-01-05

**Authors:** Hehua Zhang, Yang Xia, Xiangsu Zhang, Qing Chang, Yuhong Zhao

**Affiliations:** ^1^Clinical Research Center, Shengjing Hospital of China Medical University, Shenyang, China; ^2^Department of Clinical Epidemiology, Shengjing Hospital of China Medical University, Shenyang, China; ^3^International Education School, China Medical University, Shenyang, China

**Keywords:** nutrition, air pollution, gestational diabetes mellitus, interaction, birth cohort

## Abstract

**Background:**

Nutritional management is the cornerstone of gestational diabetes mellitus (GDM) prevention. High quality instead of low quantity of carbohydrate intake has been paying attention in controlling glycemia. Air pollution exposure can be interacted with dietary sourced nutrients, which may modify the associations with GDM. This study aims to explore the associations between overall quality of carbohydrate intake and GDM as well as the modifying effect of prenatal air pollution exposure.

**Methods:**

Carbohydrate quality index (CQI) was calculated was calculated by summing scores of the four components; Land use regression prediction models were used to assess the air pollution exposure levels. GDM definition was based on 75 g glucose tolerance test results. Associations between pre-pregnancy CQI, pre-natal air pollution as well as the modifying effect on GDM were explored based on a birth cohort in China.

**Results:**

A total of 3,183 participants were included, of which 784 (24.63%) were diagnosed with GDM. Higher pre-pregnancy CQI was associated with a lower incidence of GDM [odds ratio (OR) = 0.75, 95% confidence interval (CI): 0.56–0.99, *P*_for trend_ = 0.04], especially for higher fasting blood glucose related GDM (OR = 0.66, 95% CI: 0.47, 0.91). Higher air pollution exposure before and during pregnancy was associated with a greater risk of GDM. Higher exposure to particulate matter with an aerodynamic diameter of < 2.5 μm (*P*_for interaction_ < 0.01), particulate matter with an aerodynamic diameter of < 10 μm (*P*_for interaction_ < 0.01), and sulfur dioxide (*P*_for interaction_ = 0.02) during pregnancy decreased the beneficial effect of high pre-pregnancy CQI on GDM.

**Conclusion:**

CQI related dietary interventions pre-pregnancy to prevent GDM incidence should be considered. Women who are planning to be pregnant should avoid high exposure to air pollution during pregnancy.

## 1. Introduction

Gestational diabetes mellitus (GDM) is defined as chronic hyperglycemia during the gestational period in women without prior diabetes (1). GDM is the most common metabolic disorder during pregnancy; its average prevalence is 17% worldwide, and is higher in Southeast Asia than in North America (2). GDM is associated with elevated risk of long-term complications in mothers and infants, including polyhydramnios, stillbirth, fetal macrosomia, postnatal adiposity, and diabetes (3). Thus, preventing GDM before pregnancy is highly important for the overall health of both mothers and children.

Nutritional management is the cornerstone of GDM prevention (4, 5). An initial study has suggested that higher carbohydrate intake can increase postprandial glycemia in pregnant women (6). The American Dietetic Association recommends a carbohydrate intake of at least 175 g/d during pregnancy to control glycemia and maintain the nutrient supply for the fetus (7, 8). Many women are consuming lower carbohydrate diets (lower than 40% of total energy), even in the preconception period (9) to prevent GDM. However, this diet might be harmful for fetal growth, given the lack of micronutrient intake and the elevated exposure to maternal ketones. Recently, evidence has suggested that higher carbohydrate intake (60–70% of total energy) with high carbohydrate quality [lower glycemic index (GI) or sugars] during pregnancy can also control glycemia (10–12). A study in Australia has indicated that higher fiber intake pre-pregnancy decreases the risk of GDM (13). One carbohydrate source, like as fiber, cannot represent all the complicated effects of carbohydrate intake on GDM. Thus, an indicator representing the overall quality of carbohydrate intake is necessary. One such indicator is the carbohydrate quality index (CQI), which integrates the intake of dietary fiber, the GI, and the intake of whole grain, total grain, liquid, and solid carbohydrate. High CQI was associated with better micronutrient intake adequacy (14), and potential benefits in fetal development as well as GDM prevention. A cohort study has found that higher CQI is associated with a lower prevalence of subclinical atherosclerosis (15).

Air pollution exposure has been another global public health problem related to GDM (16–18). Interactions between air pollution exposure and dietary intake in several diseases including GDM have been reported in previous studies, possibly because both involve similar biological mechanisms, including variations in inflammation, oxidative reaction as well as methylation (19–21). Thus, air pollution exposure may influence the association between pre-pregnancy carbohydrate intake and GDM. We hypothesized that high quality carbohydrate intake pre-pregnancy might decrease the incidence of GDM, and this association might be modified by air pollution exposure levels. Thus, to provide evidence to support developing nutritional strategies for GDM prevention, we aimed to explore the associations between pre-pregnancy CQI and GDM, and the interactions between pre-pregnancy CQI and air pollution exposure before and during pregnancy on GDM.

## 2. Materials and methods

### 2.1. Participants

**FIGURE 1:**
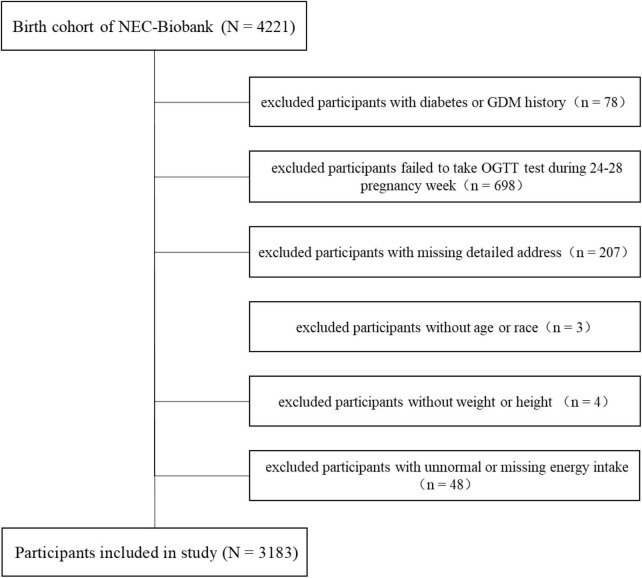
Selection process for participants.

The present study was conducted in a birth cohort belonging to the Northeast Cohort Study of China (NEC-Biobank). NEC-Biobank is a large, prospective, and dynamic cohort study focusing on typical environmental factors and chronic diseases, and covering a variety of populations including multiple races, professions, maternal-fetal, and children in Northeast China (19, 22). The protocol of the NEC-Biobank was approved by the ethics committee in Shengjing Hospital of China Medical University (no. 2017PS190K). Participants in the birth cohort were enrolled in the first or second trimester, and provided signed informed consent before inclusion. Baseline investigations included a face-to-face questionnaire survey, physical examinations, and biological sample collection started from 1st, August 2018. Follow-up was conducted in the third trimester, delivery period, and postnatal periods at 42 days, 6, and 12 months for mothers, and 12–24 months for children. By the end of August 2021, 4,221 participants were included in the birth cohort. We excluded mothers with the following conditions: history of diabetes or GDM (*n* = 78), no glucose tolerance test (OGTT) performed during weeks 24–28 of pregnancy (*n* = 698), no detailed address (*n* = 207), no age or race information (*n* = 3), missing data on weight or height (*n* = 4), unnormal (> 4,000 or < 1,500 kcal/day), or missing data regarding total energy intake per day (*n* = 48). Finally, a total of 3,183 participants were included in the analyses. The detailed selection process is shown in Figure 1.

### 2.2. CQI assessment

Dietary intake in the year before pregnancy was assessed with a validated food frequency questionnaire (FFQ) with 110 food items. Participants were asked to choose their food intake frequencies for each food item from the following seven frequency categories: “2 or 3 times per day,” “1 time per day,” “4–6 times time per week,” “2–3 times per week,” “1 time per week,” “< 1 time per week,” or “almost never.” An *ad hoc* computer program was developed to calculate the mean daily intake (g/day) of nutrients and energy according to the Chinese Food Composition Tables (23).

The CQI was used to assess the overall carbohydrate intake quality for each participant (14). Components of CQI included the whole grain/total grain ratio, dietary fiber intake (g/day), ratio of solid/total carbohydrate, and total GI. Whole grain was the sum of all types of coarse grains. Total grain referred to whole grain and refined grain products (including rice, steamed bread or steamed twisted rolls, pancakes, noodles, wontons, steamed stuffed buns or dumplings, bread, all types of porridge, and various bean vermicelli or bean starch sheets). Solid carbohydrates were calculated as the total carbohydrate minus liquid carbohydrates. Liquid carbohydrates included the carbohydrates from fruit juice and sugar sweetened beverages. The GI for each food item was calculated with the data from the FFQ combined with the Chinese Food Composition Tables (23). The total GI for each participant was calculated by first multiplying the carbohydrates consumed per food item and its specific GI, then adding the GI contributions across all food items and dividing by the total carbohydrate intake (24).

CQI was calculated by summing scores of the four components as follows: for dietary fiber intake (gram/day), ratio of solid/total carbohydrate, and GI, participants were divided into quartiles. Quartiles in the order of 1–4 were used for dietary fiber intake and the ratio of solid/total carbohydrate scores. Inverse quartiles in the order of 4–1 were used for the GI score. For the ratio of whole grain/total grain, participants who did not consume any whole grains were assigned 1 point, while the other participants were divided into two categories according to the median values, and were assigned 2 or 3 points. The CQI (the sum of the four aforementioned components) ranged from 4 to 15. Participants with higher quality of carbohydrate intake had higher CQI (Table 1). We divided participants into three categories according to three 4-point intervals (4–7 points, 8–11 points, and 12–15 points) of CQI.

**TABLE 1:** Components and calculation formula of CQI.

Components of CQI	Scores of each component			
	1	2	3	4
Total dietary fiber (g/d)	1.368–11.124	11.136–15.343	15.356–21.202	21.225–63.320
	(*n* = 795)^a^	(*n* = 796)	(*n* = 796)	(*n* = 796)
Glycemic index	68.163–81.312	64.340–68.162	60.353–64.331	33.863–60.352
	(*n* = 796)	(*n* = 796)	(*n* = 796)	(*n* = 795)
Ratio of whole grains/total grains	0	0.003–0.180	0.180–0.744	
	(*n* = 717)	(*n* = 1,463)	(*n* = 1,003)	
Ratio of solid/total carbohydrates	0.647–0.967	0.967–0.984	0.984–0.995	0.995–1
	(*n* = 795)	(*n* = 796)	(*n* = 528)	(*n* = 1,064)

CQI, carbohydrate quality index.

^a^Range of original values (counts of participants in this range; all such values).

### 2.3. Air pollution exposure assessment

Land use regression prediction models were used to assess the air pollution exposure levels to particulate matter with an aerodynamic diameter of < 2.5 μm (PM_2.5_), particulate matter with an aerodynamic diameter of < 10 μm (PM_10_), sulfur dioxide (SO_2_), nitrogen dioxide (NO_2_), carbon monoxide (CO), and ozone (O_3_) for each participant before and during pregnancy. Monthly prediction models during 2017–2021 were developing by using monthly averaged air pollution data derived from 78 national monitoring stations in Liaoning province. The detailed development process has been described elsewhere (25). Personal exposure levels to the six aforementioned air pollutants were calculated as follows. First, ArcGIS version 10.5 was used to interpolate monthly spatial distributions of each air pollutants calculated from prediction models. Second, each participant’s detailed address was transformed into latitude and longitude data. Third, the average concentration of each air pollutant was calculated according to the last menstrual period; pre-pregnancy air pollution exposure was the average over the 12 months before the last menstrual period; and air pollution exposure during pregnancy was the average over 6 months (the first and second trimesters) after the last menstrual period. If a participant changed her address in the year before pregnancy, then both addresses and the time spent at each address were recorded, and the final exposures were calculated as the average exposures at both addresses.

### 2.4. GDM definition

The 75 g OGTT during the gestational period of 24–28 weeks was a routine prenatal examination in this study. According to the recommendations of the International Association of Diabetes and Pregnancy Study Groups in 2010 (26), GDM was diagnosed when at least one of the following was met: i, fasting blood glucose level > 5.1 mmol/L; ii, blood glucose level after oral administration of 75 g of glucose for 1 h > 10 mmol/L; iii, blood glucose level after oral administration of 75 g glucose for 2 h > 8.5 mmol/L.

### 2.5. Other variables

A face to face questionnaire survey was used to collect pre-pregnancy information including the following variables: age, education level, race, family income, occupation, pre-pregnancy smoking, and alcohol consumption (yes or no), number of births, self-reported personal and family history of diabetes or GDM, personal history of other chronic diseases (including cardiac diseases, hypertension, depression, eclampsia, and polycystic ovarian syndrome), and household cooking behavior (almost none, one to two times per week, or more than three times per week). Education level was categorized as high (college graduate or above) or low (high school, middle school, or primary school graduate, or illiterate). Race was categorized as Han ethnicity or others. Occupation was divided into four categories [professional or civil servant, unskilled laborer, no formal occupation, or other (including student, farmer, etc.)]. Parity was categorized as primipara or multipara according to number of births. Comorbidity status was categorized as “no” (no personal history of other chronic diseases) or “yes” (personal history of other chronic diseases including hypertension, depression, anxiety, preeclampsia, etc.). Pre-pregnancy body mass index (BMI, kg/m^2^) was calculated as weight/(height height). The average metabolic equivalent hours per week (METs-h/week) for the total activities was calculated based on data collected from the Chinese version of the Pregnancy Physical Activity Questionnaire (27).

### 2.6. Statistical analysis

Participants were divided into three categories according to CQI (4–7 points, 8–11 points, or 12–15 points). Continuous variables are reported as mean values with standard deviations. Categorical variables are presented as counts and percentages. *P*-values for trends across the three groups of participants were examined with linear regression or logistic regression models. Participants’ characteristics were also described according to GDM status. Variance analysis and chi-squared test were used to examine continuous variables and categorized variables, respectively. Multiple logistic regression was first used to estimate the risk of GDM for each 4-point increase in CQI and was adjusted for age, BMI, race, family income, education level, occupation, family history, comorbidities, parity, smoking, and alcohol consumption, physical activity, daily intake of total energy, protein and fat in the final model. *P*-values for trends were also calculated. Multiple logistic regression was then used to estimate the risk of GDM for each inter-quartile range (IQR) increase in each air pollutant, and was adjusted for age, BMI, race, family annual income, education level, occupation, family history, comorbidities, parity, smoking, and alcohol consumption, physical activity, and household cooking behavior in the final model. The modifying effect of air pollution on CQI and GDM was examined by adding the interaction terms of each pollutant and CQI categories in the models. For significant interactions, we further assessed the risks of GDM associated with CQI according to the quartiles of each exposure level of air pollutants. For sensitive analysis, multiple linear regression was conducted to examine the linear associations among CQI, air pollution exposure, and glucose levels at fasting, and 1 and 2 h after oral administration of 75 g of glucose. The associations among the four components of CQI and GDM were also examined. SAS version 9.4 for Windows was used to perform all statistical analyses (SAS Institute Inc., Cary, NC, USA). Findings at *P* < 0.05 were considered significant.

## 3. Results

### 3.1. Descriptive statistics

This study included 3,183 participants, 784 (24.63%) of whom were diagnosed with GDM. Participant characteristics according to the three categories of CQI are shown in Table 2. Participants with higher CQI tended to be older (*P* < 0.0001), and to have higher education levels (*P* = 0.01), and higher daily intake of total energy (*P* < 0.0001), protein (*P* < 0.0001), and fat (*P* < 0.0001). Participants’ characteristics according to GDM status are shown in Supplementary Table 1. Participants with GDM tended to be older (*P* < 0.0001), and to have higher BMI (*P* < 0.0001), CQI (*P* < 0.01), pre-pregnancy air pollution exposure to PM_10_ (*P* = 0.01), and NO_2_ (*P* = 0.02), and a family history of diabetes or GDM (*P* < 0.01) or other comorbidities (*P* < 0.01).

**TABLE 2:** Characteristics of participants according to CQI categories.

Characteristics	Carbohydrate quality index			*P* for trend^b^
	4–7 points	8–11 points	12–15 points	
	(*n* = 681)	(*n* = 1,683)	(*n* = 819)	
GDM (n, percentage)	172 (25.26)	435 (25.85)	177 (21.61)	0.08
Age, years	30.25 (3.59)^a^	30.77 (3.72)	31.2 (3.82)	<0.0001
Race, Han, *n* (%)	569 (83.55)	1,374 (81.64)	680 (83.03)	0.85
BMI, kg/m^2^	22.69 (3.87)	22.62 (4.1)	22.33 (3.86)	0.08
Higher education level, *n* (%)	360 (52.86)	965 (57.34)	485 (59.22)	0.01
**Occupation, *n* (%)**				
Professionals or civil servant	235 (34.51)	657 (39.04)	314 (38.34)	0.15
Unskilled laborer	182 (26.73)	436 (25.91)	229 (27.96)	0.55
No formal occupation	161 (23.64)	372 (22.10)	183 (22.34)	0.58
Other (including student, farmer, etc.)	103 (15.12)	218 (12.95)	93 (11.36)	0.03
Family income, 10,000 yuan	11.9 (8.97)	12.57 (11.31)	12.31 (9.87)	0.5
Smoking pre-pregnancy, *n* (%)	24 (3.52)	45 (2.67)	19 (2.32)	0.17
Alcohol consumption pre-pregnancy, *n* (%)	149 (21.88)	324 (19.25)	150 (18.32)	0.09
Primipara, *n* (%)	14 (2.06)	26 (1.54)	17 (2.08)	0.92
Family history, *n* (%)	14 (2.06)	48 (2.85)	12 (1.47)	0.37
Comorbidities, *n* (%)	21 (3.08)	98 (5.82)	32 (3.91)	0.59
Total energy intake, kcal/day	1231.4 (293.28)	1577.18 (480.5)	1952.76 (622.14)	<0.0001
Protein intake, g/day	45.49 (13.01)	60.83 (19.87)	79.7 (27.59)	<0.0001
Fat intake, g/day	29.38 (10.32)	41.85 (15.98)	55.87 (21.42)	<0.0001
Physical activity, METs × h/week	142.51 (70.97)	148.57 (78.68)	148.63 (88.28)	0.16
**Air pollution exposure before pregnancy**				
PM_2.5_, μg/m^3^	38.28 (4.00)	38.59 (3.82)	38.63 (3.85)	0.10
PM_10_, μg/m^3^	68.85 (7.60)	69.45 (7.4)	69.54 (7.43)	0.09
SO_2_, μg/m^3^	22.3 (4.76)	22.38 (4.67)	22.31 (4.80)	0.98
NO_2_, μg/m^3^	32.79 (2.99)	33.07 (2.92)	33.1 (3.06)	0.05
CO, μg/m^3^	911.83 (69.62)	911.17 (67.68)	907.09 (68.18)	0.17
O_3_, μg/m^3^	108.49 (5.85)	108.63 (5.53)	108.69 (5.44)	0.5
**Air pollution exposure during pregnancy**				
PM_2.5_, μg/m^3^	41.56 (14.04)	42.36 (14.03)	40.72 (13.75)	0.18
PM_10_, μg/m^3^	78.42 (21.93)	79.19 (21.61)	76.58 (21.35)	0.07
SO_2_, μg/m^3^	20.55 (6.43)	21.15 (6.99)	20.23 (6.75)	0.26
NO_2_, μg/m^3^	34.97 (7.72)	35.72 (7.58)	34.8 (7.57)	0.53
CO, μg/m^3^	888.6 (141.06)	897.18 (136.74)	880.92 (134.47)	0.21
O_3_, μg/m^3^	107.43 (30.95)	104.59 (30.81)	106.31 (30.69)	0.57
**Household cooking frequency, *n* (%)**				
More than three times per week	211 (30.98)	491 (29.17)	247 (30.16)	0.77
One to two times per week	160 (23.49)	411 (24.42)	212 (25.89)	0.28
Less than one time per week	310 (45.53)	781 (46.41)	36 (43.95)	0.12

GDM, gestational diabetes mellitus; BMI, body mass index.

^a^Mean (standard deviation) for continuous variables and counts (percentages) for categorized variables.

^b^*P* for trend values from unadjusted logistic (for categorized variables) or linear regression (continuous variable) models.

### 3.2. Associations between pre-pregnancy CQI and GDM

Table 3 shows the associations between pre-pregnancy CQI and GDM. Higher pre-pregnancy CQI was associated with a lower incidence of GDM (*P* = 0.04). Compared with the lowest CQI category, participants in the highest category had the lowest risk of GDM (OR = 0.75, 95% CI: 0.56–0.99).

**TABLE 3:** Associations between pre-pregnancy CQI and GDM.

	Carbohydrate quality index			*P* for trend^b^
	4–7 points	8–11 points	12–15 points	
Crude model	Ref	1.01 (0.82, 1.25)^a^	0.81 (0.63, 1.03)	0.07
Model 1^c^	Ref	1.01 (0.82, 1.25)	0.77 (0.60, 0.99)	0.03
Model 2^d^	Ref	0.99 (0.8, 1.23)	0.77 (0.6, 0.99)	0.03
Model 3^e^	Ref	0.96 (0.77, 1.21)	0.75 (0.56, 0.99)	0.04

^a^ORs and 95% CIs calculated from multiple logistic regressions.

^b^Multiple logistic regression.

^c^Adjusted for age and BMI.

^d^Further adjusted for race, family income, education level, occupation, family history of diabetes or GDM, comorbidities, parity, physical activity, smoking and alcohol consumption, based on model 1.

^e^Further adjusted for daily intake of total energy, protein, and fat based on model 2.

### 3.3. Associations between air pollution exposure and GDM

Associations between each IQR increase in air pollutants before and during pregnancy and the risk of GDM are presented in Table 4. Higher air pollution exposure to PM_2.5_ (OR = 1.28, 95% CI: 1.11, 1.47), PM_10_ (OR = 1.31, 95% CI: 1.13, 1.51), SO_2_ (OR = 1.30, 95% CI: 1.13, 1.49), and NO_2_ (OR = 1.41, 95% CI: 1.20, 1.65) before pregnancy, and air pollution exposure to PM_2.5_ (OR = 1.27, 95% CI: 1.09, 1.47), PM_10_ (OR = 1.18, 95% CI: 1.02, 1.36), NO_2_ (OR = 1.29, 95% CI: 1.11, 1.50), and CO (OR = 1.17, 95% CI: 1.03, 1.33) during pregnancy, were associated with greater risk of GDM. Both higher air pollution exposure to O_3_ before (OR = 0.87, 95% CI: 0.77, 0.99) and during (OR = 0.73, 95% CI: 0.62, 0.86) pregnancy were associated with greater risk of GDM.

**TABLE 4:** Associations between each IQR increase in air pollutants and GDM.

	Air pollution exposure	IQR, μ g/m^3^	Crude model	Model1^b^	Model2^c^
Pre-pregnancy	PM_2.5_	6.30	1.29 (1.13, 1.48)^a^	1.28 (1.12, 1.47)	1.28 (1.11, 1.47)
	PM_10_	12.55	1.32 (1.15, 1.52)	1.31 (1.14, 1.51)	1.31 (1.13, 1.51)
	SO_2_	7.60	1.25 (1.09, 1.43)	1.26 (1.11, 1.45)	1.30 (1.13, 1.49)
	NO_2_	5.45	1.37 (1.18, 1.60)	1.38 (1.18, 1.61)	1.41 (1.20, 1.65)
	CO	70.00	1.00 (0.92, 1.09)	1.01 (0.93, 1.10)	1.01 (0.93, 1.11)
	O_3_	8.42	0.86 (0.76, 0.97)	0.87 (0.77, 0.99)	0.87 (0.77, 0.99)
During pregnancy	PM_2.5_	24.30	1.21 (1.05, 1.39)	1.24 (1.07, 1.43)	1.27 (1.09, 1.47)
	PM_10_	36.13	1.14 (1.00, 1.31)	1.16 (1.01, 1.34)	1.18 (1.02, 1.36)
	SO_2_	9.57	1.05 (0.94, 1.18)	1.07 (0.95, 1.20)	1.07 (0.95, 1.21)
	NO_2_	13.35	1.23 (1.06, 1.42)	1.25 (1.08, 1.45)	1.29 (1.11, 1.50)
	CO	194.47	1.11 (0.99, 1.25)	1.14 (1.01, 1.28)	1.17 (1.03, 1.33)
	O_3_	56.28	0.79 (0.68, 0.92)	0.78 (0.67, 0.91)	0.73 (0.62, 0.86)

^a^ORs and 95% CIs calculated from multiple logistic regressions.

^b^Adjusted for age and BMI.

^c^Further adjusted for race, family income, education level, occupation, family history of diabetes or GDM, comorbidities, parity, smoking and alcohol consumption, physical activity, and household cooking based on model 1.

### 3.4. Modifying effect of air pollution on CQI and GDM

**FIGURE 2:**
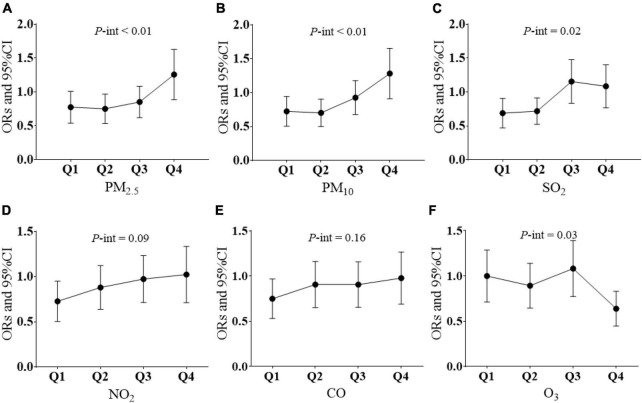
Modifying effect of air pollution on CQI and GDM. **(A)** Association between every 4-point CQI increase and GDM according to four quantiles of PM_2.5_ exposure levels. **(B)** Association between every 4-point CQI increase and GDM according to four quantiles of PM_10_ exposure levels. **(C)** Association between every 4-point CQI increase and GDM according to four quantiles of SO_2_ exposure levels. **(D)** Association between every 4-point CQI increase and GDM according to four quantiles of NO_2_ exposure levels. **(E)** Association between every 4-point CQI increase and GDM according to four quantiles of CO exposure levels. **(F)** Association between every 4-point CQI increase and GDM according to four quantiles of O_3_ exposure levels. P-int, *P*-value for interaction. Multiple logistic regression models were adjusted for age; BMI, race family income; education level; occupation; family history of diabetes or GDM; comorbidities; parity; smoking and alcohol consumption; physical activity; daily intake of total energy, protein, and fat; and household cooking; Q1, the first quartile; Q2, the second quartile; Q3, the third quartile; Q4, the forth quartile; *P*-int, *P*-value for interaction.

Interaction analysis showed that pre-pregnancy air pollution exposure had no modulatory effects on CQI and GDM. Significant interaction effects between pre-pregnancy carbohydrate quality intake and air pollution exposure to PM_2.5_ (*P*_for interaction_ < 0.01), PM_10_ (*P*_for interaction_ < 0.01), SO_2_ (*P*_for interaction_ = 0.02), and O_3_ (*P*_for interaction_ = 0.03) during pregnancy were found with GDM. Analysis results stratified by air pollution level during pregnancy (classified into four quantiles) are presented in Figure 2. Higher exposure to PM_2.5_, PM_10_, and SO_2_ during pregnancy decreased the beneficial effect of high CQI pre-pregnancy on GDM.

### 3.5. Sensitive analysis

As shown in Table 5, higher CQI was associated with only lower blood fasting glucose levels, but not 1 and 2-h glucose levels after oral administration of 75 g of glucose. We further used fasting glucose levels (≥ 5.1 mmol/L) to define GDM (fGDM). The associations between CQI in three categories and fGDM are shown in Supplementary Table 2, which indicate the risk of fGDM decreased as the CQI increased. Separate associations between scores of the four components of CQI and GDM are shown in Supplementary Table 3. Higher whole grain intake was significantly associated with lower risk of GDM (OR = 0.68, 95% CI: 0.52, 0.88; *P* for trend < 0.01). Association between air pollution exposure and blood glucose levels are shown in Supplementary Tables 4-1, 4-2. Every IQR increase in PM_2.5_, NO_2_, CO, and O_3_ during pregnancy was significantly associated with the fasting blood glucose level (PM_2.5_: β = 0.05, 95%CI: 0.01, 0.09; NO_2_: β = 0.07, 95%CI: 0.03, 0.11; CO: β = 0.04, 95%CI: 0.01, 0.08; O3: β = −0.11, 95%CI: −0.15, −0.07). We further analyzed the interaction effects between air pollution exposure during pregnancy and CQI on fGDM. The interaction *P*-values were 0.02, 0.03, < 0.01, 0.06, 0.02, and 0.01 for PM_2.5_ × CQI, PM_10_ × CQI, SO_2_ × CQI, NO_2_ × CQI, CO × CQI, and O_3_ × CQI, respectively.

**TABLE 5:** Associations between CQI and blood glucose levels at different time points during OGTT.

	GLU0	GLU1	GLU2
Crude model	−0.032 (−0.055, −0.009)^a^	−0.032 (−0.126, 0.063)	0.046 (−0.027, 0.118)
Model 1^b^	−0.034 (−0.057, −0.011)	−0.054 (−0.147, 0.039)	0.022 (−0.049, 0.094)
Model 2^c^	−0.033 (−0.056, −0.01)	−0.052 (−0.146, 0.041)	0.024 (−0.047, 0.096)
Model 3^d^	−0.034 (−0.06, −0.008)	−0.08 (−0.187, 0.027)	0.024 (−0.058, 0.106)

GLU0: fasting blood glucose level before oral administration of 75 g of glucose; GLU1: blood glucose level 1 h after oral administration of 75 g of glucose; GLU2: blood glucose level 2 h after oral administration of 75 g of glucose.

^a^β-value and 95%CI from multiple linear regressions.

^*b*^Adjusted for age and BMI.

^*c*^Further adjusted for race, family income, education level, occupation, family history of diabetes or GDM, comorbidities, parity, physical activity, smoking and alcohol consumption, based on model 1.

^*d*^Further adjusted for daily intake of total energy, protein and fat based on model 2.

## 4. Discussion

### 4.1. Main findings

Our findings indicated that higher pre-pregnancy CQI decreased the risk of GDM, particularly fasting blood glucose. Higher air pollution exposure to PM_2.5_, PM_10_, and SO_2_ during pregnancy decreased the protective effect of high carbohydrate intake quality on GDM. Higher O_3_ exposure during pregnancy strengthened the protective effect of high carbohydrate intake quality on GDM.

### 4.2. CQI and GDM

Previous studies have gradually recognized that merely decreasing the total quantity of carbohydrate intake can have a series of adverse effects on the health and development of mothers and children, instead of controlling current blood glucose levels (8, 9, 28). Increasing attention is being paid to the quality or nutritional source of carbohydrate intake (29, 30). A recent systematic review has indicated that higher quality carbohydrate intake during pregnancy can control blood glucose levels in GDM patients (31). An intervention experiment has indicated that oat and buckwheat compounds decrease the fasting glucose levels in diabetic rats (32). A clinical trial has reported that carbohydrate counting and adherence to the DASH diet decreases fasting blood glucose levels in GDM with metformin therapy (30). Most interventional studies have focused on dietary intake during pregnancy and GDM control (30, 33). Only limited studies have explored components of carbohydrate intake and the risk of GDM development. A study in the Seremban cohort has reported that glycemic index and glycemic load in dietary intake before pregnancy are not associated with GDM (34). A cohort study from Australia has reported that higher intake of cereal pre-pregnancy increases the risk of GDM, whereas higher intake of dietary fiber, fruits, and fruit juices decreases the risk of GDM (13). However, previous studies have usually focused on only one or two components of carbohydrate rather than overall intake quality. In this study, we took the total quality of carbohydrate intake before pregnancy into account and found a protective effect of higher pre-pregnancy CQI on GDM, particularly that related to fasting glucose levels, which is of high importance for dietary modification among women preparing for pregnancy. Regarding the components of CQI, we found that a higher ratio of whole grain intake pre-pregnancy significantly decreased the risk of GDM, in agreement with findings from a previous study (13).

### 4.3. Interactions between CQI and air pollution

Air pollution exposure before and during pregnancy has been a major environmental risk factor for GDM. The association between air pollution exposure before pregnancy and during pregnancy and GDM found in this study was in accordance with the results of previous studies (19, 35).

To our knowledge, no study has reported the interaction effect of CQI and air pollution on GDM. Studies since 2008 have focused mainly on the modifying effects of dietary or nutritional supplements on the association between air pollution exposure and chronic diseases (36). A birth cohort study in Spain has found that lower intake of vegetables and vitamin D, as well as a lack of breastfeeding, strengthen the adverse effects of NO_2_ exposure on infant mental development (37). A cohort study in the United States has found that a higher Mediterranean diet score is associated with lower rates of cardiovascular disease risk due to long-term PM_2.5_ exposure (20). Similar modification effects of vegetables, fruits, and Mediterranean diet intake have also been found on O_3_ exposure and lung function (36). Studies have also indicated that fish oil or other oil supplements mitigate the effects of inflammation in cardiovascular outcomes caused by air pollution exposure (38–40).

In this study, we examined the modifying effect of air pollution exposure on CQI and GDM, because dietary intervention is the fundamental strategy for the prevention and control of GDM. The elevated inflammatory response and oxidative stress due to higher exposure to air pollutants are associated with the development of GDM (41, 42). Higher CQI intake, with higher dietary fiber, lower glycemic index, and higher whole grain, has been found to be associated with lower GDM risk, through elevating plasma alkylresorcinols (43), slowing postprandial insulin responses (44), and decreasing oxidative stress (45). Thus, the modifying effect of air pollution on CQI and GDM might be due to that higher CQI decreased while air pollution of PM_2.5_, PM_10_ and SO_2_ increased the oxidative stress (46, 47), particulate matter induced inflammation and resulting oxidative stress has been proved to be a major pathway for the development of GDM. The finding that O_3_ exposure strengthened the interaction between CQI and GDM was unexpected, because O_3_ is a strong oxidant and can lead to insulin resistance by inducing oxidative stress. Possible reasons for this finding might be that pre-pregnancy O_3_ exposure could cause fetal loss before the GDM diagnosis is made (48) or that higher O_3_ exposure is correlated with lower exposure to all other pollutants (49) (Supplementary Table 5).

### 4.4. Limitations and strengths

This is the first study to explore the associations between pre-pregnancy CQI and GDM, as well as the modulatory effects of air pollution, in a birth cohort. This study has several limitations. First, the participants in our birth cohort mainly came from urban areas and had high education levels. The CQI in this sample was categorized into three levels because of the moderate variability in dietary intake, and the results of this study do not represent people in rural areas. Second, although the FFQ was collected by trained reviewers, we could not rule out the possibility of dietary intake recall bias before pregnancy. Third, except for household cooking behavior, we did not consider other indoor air pollution, thus potentially resulting in biases in precision air pollution exposure. Fourth, although we adjusted for many covariates, residual and unmeasured factors might have contributed to the observed associations between CQI and GDM. Fifth, longitudinal statistical and analytical results cannot be involved in this study, future longitudinal researches should be conducted to provide more strengthened evidences between CQI, air pollution and GDM.

## 5. Conclusion

High quality carbohydrate intake pre-pregnancy is associated with a decreased risk of GDM. A CQI related dietary intervention pre-pregnancy to prevent GDM incidence should be considered. However, the modification effects of air pollution during pregnancy cannot be ignored, and women who are preparing to be pregnant should avoid high exposure to PM_2.5_, PM_10_, and SO_2_ during pregnancy.

## Data availability statement

The original contributions presented in this study are included in the article/Supplementary material, further inquiries can be directed to the corresponding author.

## Ethics statement

The studies involving human participants were reviewed and approved by the Ethics Committee in Shengjing Hospital of China Medical University. The patients/participants provided their written informed consent to participate in this study.

## Author contributions

HZ and YX analyzed the cohort data and were responsibility for the integrity of the data and the accuracy of the data analysis. HZ was a major contributor in writing the manuscript. QC and XZ reviewed and revised the manuscript. YZ designed the study process. All authors read and approved the final manuscript.
