# Supplementary material for: Carbohydrate intake quality and gestational diabetes mellitus, and the modifying effect of air pollution
Source: Front Nutr. 2023 Jan 5;9:992472. doi: 10.3389/fnut.2022.992472 (PMC9849808; doi:10.3389/fnut.2022.992472)
Supplement: Supplementary file 1 [file Table_1.DOCX]

**Table S1.** Characteristics of participants according to GDM status.

| Variables | GDM | | *P* value |
| --- | --- | --- | --- |
|  | Yes (n = 784) | No (n = 2399) |  |
| Age, years | 31.69 (31.44, 31.95) | 30.46 (30.32, 30.61) | <0.0001 |
| Family income per year, 10000 yuan | 12.17 (11.42, 12.91) | 12.42 (12, 12.84) | 0.56 |
| Race, Han, n (%) | 645 (82.27) | 1978 (82.45) | 0.91 |
| BMI, kg/m^2^ | 23.43 (23.16, 23.71) | 22.27 (22.11, 22.43) | <0.0001 |
| Family history of diabetes or GDM, n (%) | 28 (3.57) | 46 (1.92) | <0.01 |
| Comorbidity, n (%) | 54 (6.89) | 97 (4.04) | <0.01 |
| Physical activity, METs ×h/week | 141.27 (135.69, 146.85) | 149.26 (146.07, 152.45) | 0.02 |
| Primipara, n (%) | 768 (97.96) | 2358 (98.29) | 0.54 |
| Occupation, n (%) |  |  |  |
| Professionals or civil servant | 288 (36.73) | 918 (38.27) | 0.44 |
| Unskilled laborer | 199 (25.38) | 648 (27.01) | 0.37 |
| No formal occupation | 195 (24.87) | 521 (21.72) | 0.07 |
| Other (including student, farmer, etc) | 102 (13.01) | 312 (13.01) | 1.00 |
| Higher education level, n (%) | 448 (57.14) | 1362 (56.77) | 0.86 |
| Pre-pregnancy smoking, n (%) | 22 (2.81) | 66 (2.75) | 0.94 |
| Pre-pregnancy alcohol drinking, n (%) | 146 (18.62) | 477 (19.88) | 0.44 |
| Total energy intake, kcal/day | 1579.3 (1540.86, 1617.73) | 1606.56 (1584.59, 1628.53) | 0.23 |
| Protein intake, g/day | 62.1 (60.42, 63.79) | 62.5 (61.54, 63.47) | 0.69 |
| Fat intake,g/day | 42.16 (40.84, 43.49) | 42.99 (42.24, 43.75) | 0.27 |
| CQI trisectional quantiles, n (%) |  |  |  |
| The first quantile (4 - 7) | 172 (21.94) | 509 (21.22) | 0.67 |
| The second quantile (8 - 11) | 435 (55.48) | 1248 (52.02) | 0.09 |
| The third quantile (12 - 15) | 177 (22.58) | 642 (26.76) | 0.02 |
| Air pollution exposure before pregnancy |  |  |  |
| PM_2.5_, μg/m^3^ | 38.98 (38.71, 39.25) | 38.39 (38.24, 38.54) | <0.001 |
| PM10, μg/m^3^ | 70.25 (69.73, 70.78) | 69.05 (68.75, 69.35) | <0.0001 |
| SO2, μg/m^3^ | 22.8 (22.46, 23.13) | 22.2 (22.01, 22.39) | 0.8 |
| NO2, μg/m^3^ | 33.39 (33.18, 33.6) | 32.9 (32.78, 33.01) | <0.0001 |
| CO, μg/m^3^ | 909.72 (904.92, 914.51) | 910.44 (907.71, 913.18) | 0.8 |
| O3, μg/m^3^ | 108.21 (107.82, 108.6) | 108.75 (108.52, 108.97) | 0.02 |
| Air pollution exposure during pregnancy |  |  |  |
| PM_2.5_, μg/m^3^ | 42.81 (41.83, 43.79) | 41.43 (40.87, 41.99) | 0.02 |
| PM10, μg/m^3^ | 79.47 (77.95, 80.98) | 77.99 (77.13, 78.86) | 0.1 |
| SO2, μg/m^3^ | 20.93 (20.45, 21.41) | 20.74 (20.47, 21.01) | 0.49 |
| NO2, μg/m^3^ | 35.92 (35.39, 36.46) | 35.13 (34.82, 35.43) | 0.01 |
| CO, μg/m^3^ | 897.55 (887.94, 907.16) | 889.07 (883.58, 894.56) | 0.13 |
| O3, μg/m^3^ | 103.01 (100.85, 105.16) | 106.5 (105.27, 107.73) | <0.01 |
| Household cooking frequency, n (%) |  |  |  |
| Over three times per week | 246 (31.38) | 703 (29.30) | 0.27 |
| One to two times per week | 182 (23.21) | 601 (25.05) | 0.3 |
| Lower then one time per week | 356 (45.41) | 1095 (45.64) | 0.91 |

Abbreviations: BMI, body mass index; GDM, gestational diabetes mellitus;

^a^ Least square mean (95% confidence interval) (all such values);

^b^ Counts (percentages) (all such values);

^c^ Analysis of variance or chi-squared test.

**Table S2.** Association between CQI and fasting glucose level (≥5.1 mmol/L) to define GDM (fGDM).

| **CQI categories** | **Crude model** | **model1** | **model2** | **model3** |
| --- | --- | --- | --- | --- |
| 4 - 7 points | ref | ref |  | ref |
| 8 - 11 points | 0.92 (0.73, 1.17) ^a^ | 0.92 (0.72, 1.17) | 0.90 (0.71, 1.15) | 0.90 (0.70, 1.16) |
| 12 - 15 points | 0.68 (0.51, 0.90) | 0.66 (0.49, 0.87) | 0.65 (0.49, 0.87) | 0.66 (0.47, 0.91) |
| *P* for trend | <0.01 | <0.01 | <0.01 | 0.01 |

^a^ ORs and 95% confidence interval from multiple logistic regressions;

model1: adjusted for age and BMI;

model2: further adjusted for race family income, education level, occupation, family history of diabetes or GDM, comorbidities, parity, smoking and alcohol drinking behaviors, physical activity based on model1;

model3: further adjusted for daily intake of total energy, protein and fat based on model2.

**Table S3.** Separate associations between the four components of CQI and GDM.

| Component of CQI | score | GDM | *P* for trend |
| --- | --- | --- | --- |
| Total dietary fiber | 1 | ref | 1 |
|  | 2 | 0.81 (0.64, 1.04) ^a^ |  |
|  | 3 | 0.96 (0.72, 1.27) |  |
|  | 4 | 0.99 (0.69, 1.41) |  |
| Ratio of solid /total carbohydrates | 1 | ref | 0.85 |
|  | 2 | 1.11 (0.87, 1.4) |  |
|  | 3 | 0.89 (0.68, 1.18) |  |
|  | 4 | 1.03 (0.82, 1.29) |  |
| Glycaemic index | 1 | ref | 0.16 |
|  | 2 | 0.92 (0.72, 1.18) |  |
|  | 3 | 0.84 (0.65, 1.09) |  |
|  | 4 | 0.85 (0.65, 1.11) |  |
| Ratio of whole grains/total grains | 1 | ref | <0.01 |
|  | 2 | 0.89 (0.72, 1.10) |  |
|  | 3 | 0.71 (0.56, 0.90) |  |

^a^ ORs and 95% confidence intervals from multiple logistic regressions. Model adjusted for age, BMI, race family income, education level, occupation, family history of diabetes or GDM, comorbidities, parity, smoking and alcohol drinking behaviors, physical activity, daily intake of total energy, protein and fat.

**Table S4-1.** Association between air pollution exposure before pregnancy and blood glucose levels.

|  | GLU0 | GLU1 | GLU2 |
| --- | --- | --- | --- |
| PM_2.5_ | 0.09 (0.06, 0.13) ^a^ | 0.15 (0.05, 0.25) | 0.17 (0.08, 0.25) |
| PM_10_ | 0.10 (0.06, 0.13) | 0.17 (0.06, 0.28) | 0.18 (0.09, 0.26) |
| SO_2_ | 0.06 (0.02, 0.10) | -0.02 (-0.12, 0.09) | 0.06 (-0.03, 0.15) |
| NO_2_ | 0.11 (0.07, 0.15) | 0.13 (0.01, 0.26) | 0.18 (0.08, 0.28) |
| CO | 0.00 (-0.02, 0.02) | 0.00 (-0.07, 0.06) | 0.01 (-0.04, 0.07) |
| O_3_ | -0.05 (-0.08, -0.01) | -0.05 (-0.15, 0.04) | -0.07 (-0.15, 0.01) |

GLU0: fasting blood glucose level before oral administration of 75-g of glucose; GLU1: blood glucose level in one hour after oral administration of 75-g of glucose; GLU2: blood glucose level in two hours after oral administration of 75-g of glucose.

^a^β value and 95%CI from multiple linear regressions. Models adjusted for age, BMI, race, family income, education level, occupation, family history of diabetes or GDM, comorbidities, parity, physical activity, smoking and alcohol drinking behaviors, and household cooking.

**Table S4-2.** Association between air pollution exposure during pregnancy and blood glucose levels.

|  | GLU0 | GLU1 | GLU2 |
| --- | --- | --- | --- |
| PM_2.5_ | 0.05 (0.01, 0.09) ^a^ | -0.10 (-0.21, 0.02) | -0.03 (-0.12, 0.06) |
| PM_10_ | 0.03 (-0.01, 0.06) | -0.12 (-0.22, -0.01) | -0.05 (-0.14, 0.03) |
| SO_2_ | 0.02 (-0.01, 0.05) | -0.02 (-0.1, 0.07) | -0.01 (-0.08, 0.06) |
| NO_2_ | 0.07 (0.03, 0.11) | 0.04 (-0.16, 0.07) | -0.01 (-0.1, 0.08) |
| CO | 0.04 (0.01, 0.08) | -0.01 (-0.10, 0.08) | 0.00 (-0.08, 0.07) |
| O_3_ | -0.11 (-0.15, -0.07) | 0.03 (-0.09, 0.16) | -0.03 (-0.13, 0.08) |

GLU0: fasting blood glucose level before oral administration of 75-g of glucose; GLU1: blood glucose level in one hour after oral administration of 75-g of glucose; GLU2: blood glucose level in two hours after oral administration of 75-g of glucose.

^a^β value and 95%CI from multiple linear regressions. Models adjusted for age, BMI, race, family income, education level, occupation, family history of diabetes or GDM, comorbidities, parity, physical activity, smoking and alcohol drinking behaviors, and household cooking.

**Table S5.** Correlation analysis between different exposure levels of air pollutants during pregnancy

|  | PM_2.5_ | PM_10_ | SO_2_ | NO_2_ | CO | O_3_ |
| --- | --- | --- | --- | --- | --- | --- |
| PM_2.5_ | 1 | 0.94 ^a^ | 0.74 | 0.83 | 0.64 | -0.7 |
| PM_10_ | 0.94 | 1 | 0.71 | 0.73 | 0.44 | -0.51 |
| SO_2_ | 0.74 | 0.71 | 1 | 0.84 | 0.6 | -0.67 |
| NO_2_ | 0.83 | 0.73 | 0.84 | 1 | 0.81 | -0.85 |
| CO | 0.64 | 0.44 | 0.6 | 0.81 | 1 | -0.81 |
| O_3_ | -0.7 | -0.51 | -0.67 | -0.85 | -0.81 | 1 |

^a^ Spearman correlation Coefficients.

All *P* values were lower than 0.0001.
